# Supplementary material for: The Effectiveness and Harms of PSA-Based Prostate Cancer Screening: A Systematic Review
Source: Healthcare (Basel). 2025 Jun 9;13(12):1381. doi: 10.3390/healthcare13121381 (PMC12192897; doi:10.3390/healthcare13121381)
Supplement: Supplementary file 1 [file healthcare-13-01381-s001.zip › healthcare-3599865-supplementary.pdf]

Table S1. Risk of Bias Table for 16 Included Studies.

Study	Design	Random Sequence	Allocation	Blinding (Participants)	Blinding (Outcome)	Incomplete Data	Selective Reporting	Conflict of Interest
ProScreen Trial [16]	RCT	Low	Low	Unclear	Low	Low	Low	Low
MRI Screening Meta-analysis [17]	Meta-analysis	N/A	N/A	N/A	Low	Low	Low	Low
Biomarker vs MRI [18]	RCT	Low	Low	Unclear	Low	Low	Low	Low
Stockholm3 Cohort [19]	Simulation model	N/A	N/A	Unclear	Unclear	Low	Low	Low
MRI vs TRUS Biopsy [20]	RCT	Low	Low	Unclear	Low	Low	Low	Low
AUA Guideline [21]	Guideline	N/A	N/A	N/A	N/A	N/A	Low	Low
Policy Review [22]	Review	N/A	N/A	N/A	Unclear	N/A	Low	Low
Screening Policy Review [23]	Review	N/A	N/A	N/A	Unclear	N/A	Low	Low
USPSTF Guideline [24]	Guideline	N/A	N/A	N/A	N/A	N/A	Low	Low
USPSTF Systematic Review [25]	Systematic Review	N/A	N/A	N/A	Low	Low	Low	Low
CAP Trial [26]	RCT	Low	Low	Unclear	Low	Low	Unclear	Low
ERSPC Trial [27]	RCT	Low	Low	Unclear	Low	Low	Low	Low
PSA Meta-analysis [28]	Meta-analysis	N/A	N/A	N/A	Low	Low	Low	Low
Cancer Overdiagnosis [29]	Ecological	N/A	N/A	N/A	Unclear	N/A	Low	Low
PSA & Mortality (Korea) [30]	Cohort	N/A	N/A	Unclear	Unclear	Low	Low	Low
ERSPC 13-Year [31]	Cohort	Low	Low	Unclear	Low	Low	Low	Low
